# Real-world clinical outcomes of apalutamide versus abiraterone with androgen deprivation therapy for metastatic hormone-sensitive prostate cancer

**DOI:** 10.1007/s11096-025-01920-4

**Published:** 2025-05-06

**Authors:** Eduardo Pons-Fuster, Celia Maria Gonzalez-Ponce, Silverio Ros-Martinez, Juan José Fernández-Ávila, María Sacramento Díaz-Carrasco, Alberto Espuny-Miró

**Affiliations:** 1 Clinical Pharmacy and Therapeutics Research Group, Servicio de Farmacia Hospitalaria, Virgen de la Arrixaca University Clinical Hospital (HCUVA), Ctra. Madrid-Cartagena, s/n, 30120 El Palmar, Spain; 2 Clinical and Translational Oncology Research Group, Clinical Oncology Unit, Virgen de la Arrixaca University Clinical Hospital (HCUVA), El Palmar, Spain

**Keywords:** Abiraterone, Antineoplastic agents, Apalutamide, Prostate-specific antigen, Prostatic neoplasms

## Abstract

**Background:**

Metastatic hormone-sensitive prostate cancer (mHSPC) is an aggressive disease with a poor prognosis. Current treatment guidelines recommend combining androgen receptor axis-targeted therapies (ARATs) with androgen deprivation therapy (ADT) for mHSPC. While individual ARATs have shown success, few studies directly compare their effects.

**Aim:**

To compare the safety and clinical outcomes of abiraterone acetate (abiraterone) and apalutamide in chemotherapy-naïve mHSPC patients, focusing on prostate-specific antigen (PSA) kinetics, safety, and survival outcomes.

**Method:**

A retrospective, single-centre study included 107 chemotherapy-naïve mHSPC patients treated with abiraterone or apalutamide plus ADT. PSA levels were measured at baseline and during treatment. Primary outcomes were PSA progression-free survival (PSA-PFS) and overall survival (OS). Adverse events were recorded. Inverse probability treatment weighting adjusted baseline differences.

**Results:**

Median PSA-PFS significantly favoured apalutamide (log-rank *p* = 0.015). Achieving PSA ≤ 0.02 ng/mL was strongly associated with delayed progression (HR 0.07, 95% CI 0.02–0.28; *p* < 0.001). OS did not differ significantly between groups (*p* = 0.504). Apalutamide achieved lower median nadir PSA (0.02 ng/mL vs. 0.23 ng/mL, *p* < 0.001) and shorter mean time to nadir (4.5 vs. 7.2 months, *p* = 0.001), with more patients reaching ultralow PSA levels (≤ 0.02 ng/mL) during follow-up. Adverse events occurred more frequently with apalutamide (71.2% vs. 46.5%, *p* = 0.015), with fatigue and rash being the most common.

**Conclusion:**

Apalutamide demonstrated deeper and more sustained PSA reductions, translating into delayed disease progression compared to abiraterone. Both treatments were generally well tolerated, though adverse events were more prevalent with apalutamide.

**Supplementary Information:**

The online version contains supplementary material available at 10.1007/s11096-025-01920-4.

## Impact statements

- Apalutamide showed greater effectiveness in achieving sustained PSA reductions and delaying disease progression compared to abiraterone in chemotherapy-naïve mHSPC patients over a 12-month follow-up.
- Achieving ultralow PSA levels (≤ 0.02 ng/mL) was strongly associated with prolonged progression-free survival, highlighting the importance of ultralow PSA levels in predicting treatment success.
- Increased adverse events, particularly fatigue and rash, in patients receiving apalutamide emphasise the need for proactive management strategies to enhance tolerability and adherence.

## Introduction

Metastatic hormone-sensitive prostate cancer (mHSPC) is an aggressive disease impacting multiple organs and systems, resulting in a poor prognosis [1]. Current treatment guidelines, based on clinical trial evidence, recommend using androgen receptor axis-targeted therapies (ARATs) in combination with androgen deprivation therapy (ADT) for patients with mHSPC [2–5]. This combination has been shown to decline prostate specific-antigen (PSA) levels, delay disease progression and extend survival compared to ADT alone [6–10]. The LATITUDE trial showed that adding abiraterone acetate (hereafter referred to as abiraterone) and prednisone to ADT significantly improved radiological progression-free survival (rPFS), PSA progression-free survival (PSA-PFS), and overall survival (OS) compared to ADT plus placebo [6, 9]. Similarly, the TITAN trial demonstrated that apalutamide combined with ADT led to improved rPFS, PSA-PFS, and OS compared to ADT plus placebo [7, 8]. Thus, the significant role of ARATs in mHSPC emphasises the need for a thorough understanding of their relative safety and effectiveness to refine treatment approaches.

Low PSA levels achieved with combination therapy are strongly associated with improved outcomes in mHSPC [10, 11]. Advances in ultrasensitive PSA assays have increasingly enabled the detection of levels below 0.2 ng/mL, referred to as ultralow PSA (≤ 0.02 ng/mL). Recent studies indicate that achieving PSA levels below 0.02 ng/mL during combination therapy is associated with substantial and durable benefits in disease control [12, 13]. Thus, understanding how ARATs contribute to achieving and maintaining these ultralow PSA levels is essential for optimising treatment strategies in mHSPC.

Although individual therapies have shown success, few studies have directly compared the effects of ARATs in mHSPC, leaving certain clinical outcomes unclear and necessitating further investigation [13–16].

### Aim

This study evaluated and compared the clinical outcomes of abiraterone and apalutamide as first-line therapies for chemotherapy-naïve mHSPC patients in a real-world context, with a focus on PSA kinetics, safety and survival outcomes.

### Ethics approval

The study conformed to the principles of the Declaration of Helsinki and the Good Clinical Practice Guidelines, and was also approved by the Ethics Committee of the University Hospital Virgen Arrixaca (2024-9-13-HCUVA) in November 2024.

## Method

### Study design and patients

A retrospective, single-centre, observational study was conducted on patients with mHSPC who received either abiraterone or apalutamide in combination with ADT as first-line treatment from August 2017 to August 2024. Patients were excluded if they had prior or concurrent treatment with docetaxel or other ARATs. Participants were identified through institutional databases. This study adhered to the Declaration of Helsinki and Good Clinical Practice Guidelines and was approved by the local Ethics Committee in October 2024.

### Study variables

Data collection was performed using electronic medical records and the SAVAC 4.0 pharmacy database. Baseline clinical and demographic characteristics of the patients, including age, Gleason score, ECOG (Eastern Cooperative Oncology Group) status, history of prostatectomy, previous radiotherapy treatment, metastasis stage, baseline testosterone levels were documented. PSA levels were measured at baseline and subsequently at 1, 3, 6, 9, and 12 months. Adverse events were classified according to the Common Terminology Criteria for Adverse Events (CTCAE), version 5.10. Subsequent treatments were recorded.

### Assessments

PSA progression-free survival was defined as the time from therapy initiation to the first PSA increase of at least 25% and 2 ng/mL above nadir, confirmed by a second measurement at least three weeks later, following the Prostate Cancer Clinical Trials Working Group (PCWG2) criteria [17]. Overall survival was measured from the start of treatment to the date of death from any cause. Surviving patients were censored on the date of their last follow-up or at the end of the study. PSA kinetics were evaluated in both treatment groups. Key parameters assessed included changes in PSA from baseline, nadir PSA (defined as the lowest PSA level recorded, requiring a minimum of two measurements, with one taken at six months or a single test with an ultralow PSA level within 12 months), time to nadir, the percentage of patients achieving any decrease in PSA, PSA50 (50% reduction from baseline), PSA90 (90% reduction from baseline), PSA ≤ 0.2 (PSA levels ≤ 0.2 ng/mL), PSA ≤ 0.02 (PSA levels ≤ 0.02 ng/mL), time with ultralow PSA levels (defined as the duration with PSA maintained at or below 0.02 ng/mL within 12 months), PSA changes from baseline at 12 weeks, and the maximum PSA decrease observed during treatment. Safety analysis recorded adverse events occurring throughout the treatment period as documented in medical records.

### Statistical analysis

Statistical analysis was performed using R software (v4.41; R Core Team 2024). Descriptive analysis included frequency tables and percentages for categorical variables, while continuous variables were summarised using mean and standard deviation (SD) or median and interquartile range [Q1–Q3].

Inverse probability treatment weighting (IPTW) was applied to address baseline differences between patients with mHSPC starting on apalutamide versus abiraterone to enable valid comparison between treatment groups. IPTW was based on a propensity score calculated with relevant confounders at prostate cancer diagnosis (Table S1). Applying a weight to each individual subject creates a standardised pseudo-population, allowing adjusted comparisons of treatment effects while mitigating observed confounding. Standardised difference (SD) was used to assess the balance of covariates between treatment groups after applying IPTW. While a standardised difference of < 0.1 is commonly used, some authors have proposed a threshold of < 0.2 as acceptable for balance diagnostics in IPTW analyses, particularly when perfect balance in small samples is challenging [18, 19]. Therefore, we adopted a < 0.2 threshold to assess covariate balance post-weighting, ensuring that the IPTW approach effectively adjusted for confounding variables and facilitated unbiased comparisons of treatment outcomes.

PSA-PFS and OS were estimated using weighted Kaplan–Meier curves, with comparisons made via log-rank tests. Hazard ratios (HR) were calculated using the Cox proportional hazards model, with univariate analyses followed by Cox multivariate analyses to evaluate factors influencing patient prognosis.

For the PSA kinetics study, normality of data was assessed with the Shapiro–Wilk test. Statistical comparisons of the weighted population for categorical variables were conducted using Fisher’s exact test, while the Mann–Whitney U test was applied for continuous variables. For the safety analysis, no weighting was applied. Missing data were excluded, and no imputation was performed. All *p*-values < 0.05 were considered statistically significant.

## Results

### Participants’ demographics and baseline parameters

A total of 143 mHSPC patients treated with ARATs were identified in our medical databases, of which 107 were chemotherapy naïve patients treated with abiraterone (43) or apalutamide (64) as first-line treatment (Supplementary Fig. 1). After IPTW, the weighted number of patients was 39 in the abiraterone group and 65 in the apalutamide group (Table 1). Baseline patient characteristics were generally well balanced between the two weighted cohorts. Patients in the abiraterone cohort had a higher median baseline PSA level (19.3 [4.4–47.5] vs. 4.6 [1.2–37.8], SMD: 0.698, *p* = 0.033).

**Table 1:** Baseline characteristics

Variables	Non-weighted population				Weighted population			
	Abiraterone	Apalutamide	*p*	Standarised difference	Abiraterone	Apalutamide	*p*	Standarised difference
	(n = 43)	(n = 64)			(n = 39)	(n = 65)		
Age, years, mean ± SD	68.0 ± 10.7	70.7 ± 7.7	0.188	0.288	71.8 ± 10.7	71.0 ± 7.7	0.511	0.086
Follow-up time, months, median [Q1-Q3]	17.1 [9.4–34.0]	25.1 [14.8–32.7]	0.147	0.171	16.7 [6.2- 26.3]	24.8 [11.8–31.2]	0.094	0.288
Time from diagnosis to initiation of treatment, months, median [Q1-Q3]	3.7 [1.7–11.0]	28.2 [4.2–95.6]	0.001	0.723	6.12 [2.5–25.1]	14.3 [2.4–61.9]	0.325	0.198
ECOG 0, n (%)	16 (32.5)	17 (34.1)	0.962	0.055	17 (42.6)	27 (39.5)	0.96	0.063
≥ 1, n (%)	27 (67.5)	37 (64.9)			22 (57.4)	38 (60.5)		
Gleason score ≤ 7	7 (16.3)	30 (46.0)	0.003	0.678	14 (34.7)	24 (35.6)	0.94	0.018
≥ 8	36 (83.7)	34 (54.0)			25 (65.3)	41 (64.4)		
Prostatectomy, n (%)	6 (14.0)	18 (28.6)	0.126	0.363	8 (19.5)	16 (26.0)	0.54	0.156
Concomitant radiotherapy, n (%)	16 (37.2)	52 (82.5)	< 0.001	1.043	23 (59.8)	41 (65.0)	0.66	0.108
Metastasis Stage, M1a, n (%)	1 (2.3)	16 (25.4)	0.003	0.708	2 (4.0)	10 (16.4)	0.12	0.417
M1b-c, n (%)	42 (97.7)	47 (74.6)			37 (95.9)	53 (83.5)		
Synchronous metastasis, n (%)	37 (86.0)	45 (71.4)	0.126	0.363	31 (80.5)	47 (74.0)	0.54	0.156
Baseline PSA level, ng/ml, median [Q1-Q3]	37.4 [5.8–122]	6.0 [2.15–17.4]	< 0.001	1.091	19.3 [4.4–47.5]	4.6 [1.2–37.8]	0.033	0.698
Baseline testosterone level, ng/dL, median [Q1-Q3]	0.1 [0.1–0.2]	0.2 [0.1–0.4]	< 0.001	0.005	0.1 [0.1–0.2]	0.2 [0.1–0.3]	0.055	0.045

ECOG, eastern cooperative oncology group; PSA, prostate-specific antigen; SD, standard deviation

### Survival analysis

**Fig. 1:**
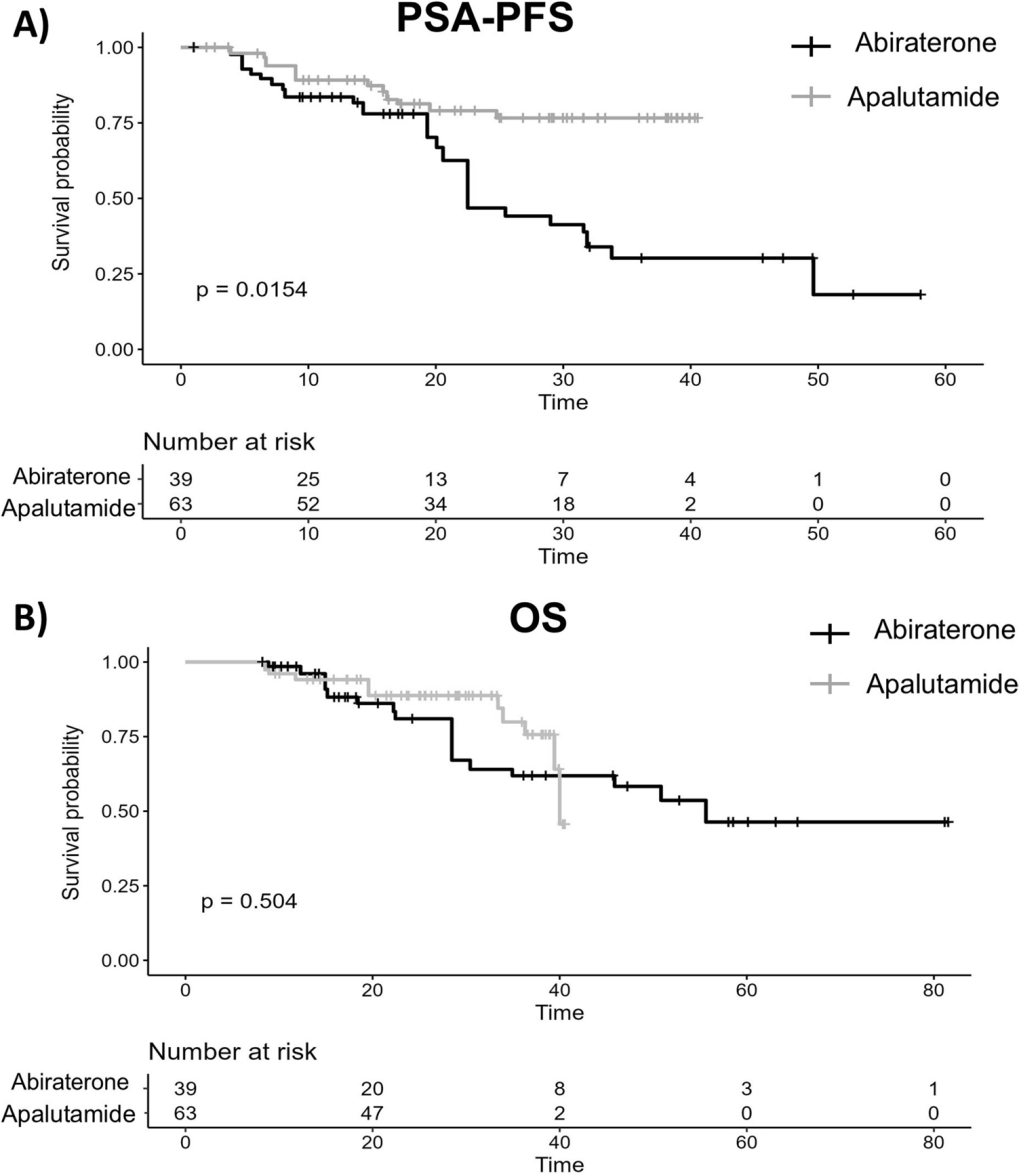
**A** PSA progression-free survival (PSA-PFS) and **B** overall survival (OS) for weighted CHPSm patients as estimated with Kaplan–Meier

Median follow-up time was 16.7 [6.2–26.3] months for abiraterone and 24.8 [11.8–31.2] months for apalutamide. During follow-up, 41% of patients in the abiraterone group and 19% of patients in the apalutamide group experienced PSA progression. The median PSA-PFS in the weighted abiraterone group was 22.5 months (95% CI, 20.1–NA), while the median PSA-PFS was not reached in the weighted apalutamide group (log-rank *p* = 0.015) (Fig. 1A). In the univariate analysis, treatment with apalutamide, as well as achieving PSA50, PSA90, PSA ≤ 0.2, and PSA ≤ 0.02 at any point during the study, significantly reduced the likelihood of PSA progression (Supplementary Table 2). In multivariate analysis, only achieving PSA ≤ 0.02 ng/mL at any point was associated with a significantly longer PSA-PFS (HR 0.07, 95% CI, 0.02–0.28; *p* < 0.001). Following disease progression, 82% of patients who started on abiraterone were treated with docetaxel, 12% switched to enzalutamide and 6% received Radium-223. In the apalutamide group, 31% were subsequently treated with docetaxel, 31% switched to abiraterone and 38% switched to enzalutamide.

For OS, 28% of patients in the abiraterone group and 17% in the apalutamide group died during the study period. No statistically significant difference in median OS was observed between the weighted groups (*p* = 0.504) (Fig. 1B).

### PSA kinetics

**Fig. 2:**
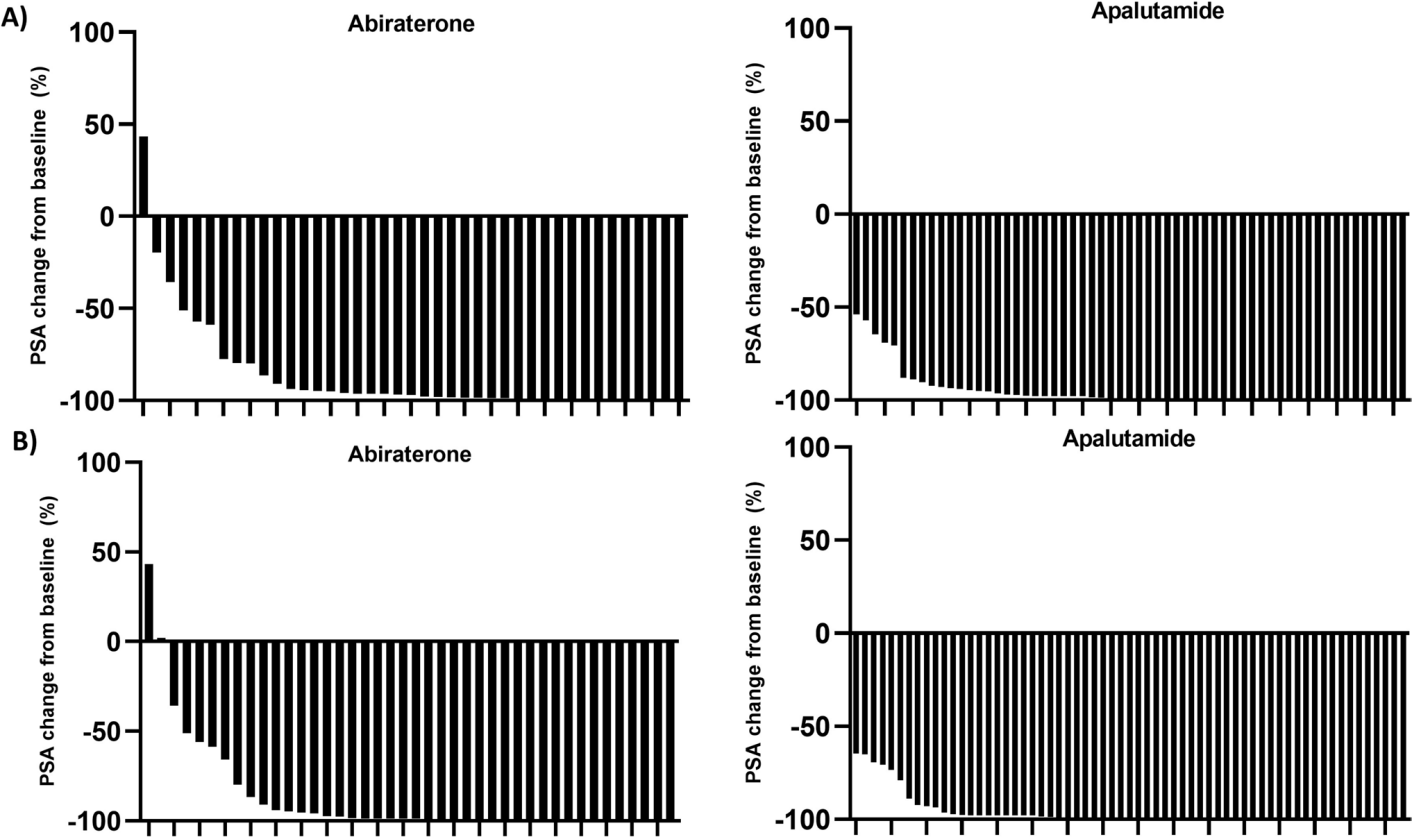
Waterfall plots of change in PSA levels at **a** 12 weeks and **b** maximum change during follow-up. PSA, prostate-specific antigen

**Fig. 3:**
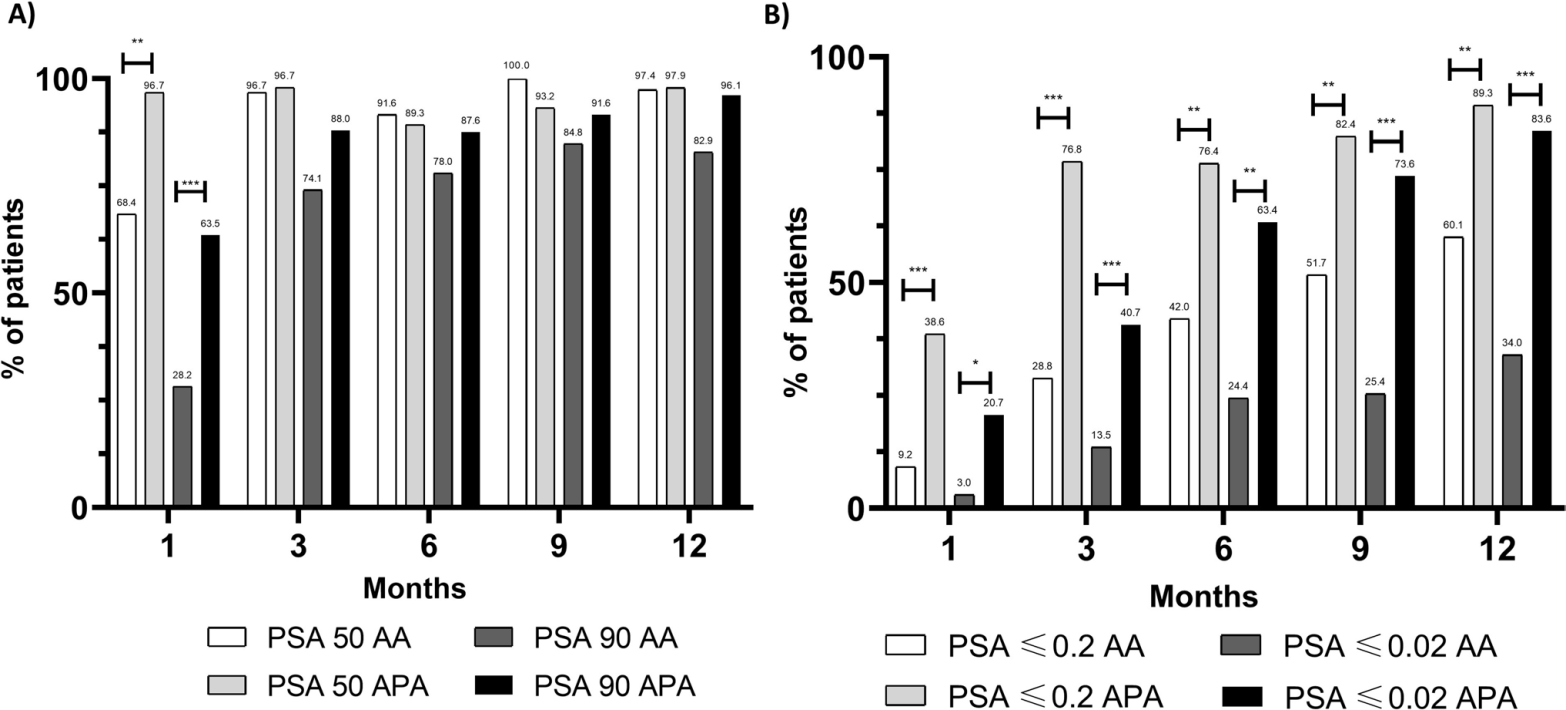
Percentage of weighted mHSPC patients achieving **a** PSA50 and PSA90 and **b** PSA < 0.2 and PSA < 0.02 levels. AA, abiraterone acetate; APA, apalutamide; PSA, prostate-specific antigen, PSA50, 50% reduction in PSA from baseline; PSA90, 90% reduction in PSA from baseline; PSA < 0.2, PSA levels ≤ 0.2 ng/mL; PSA < 0.02, PSA levels ≤ 0.02 ng/mL

Waterfall plots of changes in PSA levels at 12 weeks or maximum PSA are shown in Fig. 2. In the weighted population, apalutamide showed a lower median nadir PSA level (0.02 [0.02–0.04] vs. 0.23 [0.02–0.65] ng/ml, *p* < 0.001) and a shorter mean time to achieve PSA nadir (4.5 ± 3.3 vs. 7.2 ± 3.6 months, *p* = 0.001) compared to abiraterone (Table 2). No statistically significant differences were observed in PSA50 or PSA90 rates between treatments with the exception of the first month follow-up, where apalutamide showed higher rates in both PSA50 (96.7% vs. 68.4%, *p* < 0.01) and PSA90 (63.5% vs. 28.2%, *p* < 0.001) (Fig. 3A). A higher percentage of apalutamide patients significantly reached PSA levels ≤ 0.2 ng/mL and ≤ 0.02 ng/mL at every follow up (Fig. 3B). Finally, during the 12-month follow-up, apalutamide patients remained with ultralow PSA levels for a longer period (5.9 ± 4.2 vs. 2.1 ± 3.5 months, *p* < 0.001) than abiraterone patients.

**Table 2:** Weighted PSA kinetics

PSA kinetics	Abiraterone	Apalutamide	*p*
	(n = 39)	(n = 65)	
*Patients achieving PSA response at any time (%)*			
Any decrease	38 (98.6)	63 (100)	0.999
PSA 50	37(95.1)	60 (94.7)	0.999
PSA 90	28 (73.0)	56 (88.6)	0.080
PSA ≤ 0.2	18 (45.7)	50 (80.0)	< 0.001
PSA ≤ 0.02	9 (23.3)	43 (68.9)	< 0.001
Nadir PSA level, ng/ml, median [Q1-Q3]	0.23 [0.01–0.65]	0.02 [0.02–0.04]	< 0.001
Time to PSA Nadir, months, mean ± SD	7.2 ± 3.6	4.5 ± 3.3	0.001
Time with ultralow PSA levels (≤ 0.02 ng/mL), months, mean ± SD	2.1 ± 3.5	5.9 ± 4.2	< 0.001

PSA, prostate-specific antigen.

*p* values were calculated with Mann–Whitney’s U test

### Safety analysis

A total of 62% of patients reported adverse events during follow-up, with a higher incidence in the apalutamide group (71.2% vs. 46.5%, *p* = 0.015) (Table 3). No adverse events led to death, and only 13.8% of patients in the apalutamide group and 6.9% in the abiraterone group discontinued treatment due to adverse events. The most common adverse events in the apalutamide cohort were fatigue (35.0%), skin rash (20.3%) and hot flushes (16.6%) while oedema and arterial hypertension (11.6% each) were the most common in the abiraterone group.

**Table 3:** Adverse events

Adverse event, n (%)	Abiraterone	Apalutamide
	(n = 43)	(n = 64)
Any AE	20 (46.5)	47 (71.2)^a^
AEs leading to treatment discontinuation	3 (6.9)	9 (13.8)
GIII-GIV AEs	2 (4.2)	9 (13.8)
AEs leading to death	0 (0.0)	0 (0.0)
Fatigue GI-GII	3 (6.4)	19 (29.7)
Fatigue GIII-GIV	0 (0.0)	4 (6.2)
Hot Flushes GI-GII	2 (4.2)	11 (16.6)
Rash GI-GII	0 (0.0)	10 (15.4)
Rash GIII-GIV	0 (0.0)	3 (4.6)
Arterial Hypertension GI-GII	5 (11.6)	0 (0.0)
Hypothyroidism GI-GII	1 (2.3)	9 (14.0)
Oedema GI-GII	5 (11.6)	1 (1.5)
Diarrhea GI-GII	0 (0.0)	3 (4.6)
Myalgia GI-GII	2 (4.2)	3 (4.6)
Anorexia GI-GII	1 (1.5)	2 (3.1)
Nausea GI-GII	0 (0.0)	2 (3.1)
Urinary tract infection GI-GII	1 (2.3)	0 (0.0)
Heart failure GIII-GIV	1 (2.1)	1 (1.5)
Cognitive impairment GIII-GIV	1 (1.5)	1 (1.5)
Elevated transaminases GIII-GIV	1 (2.1)	0 (0.0)

AE, adverse events; G, grade

^a^*p* value = 0.015

## Discussion

In this study of chemotherapy-naïve mHSPC patients treated with abiraterone or apalutamide as first-line therapy in routine clinical practice, apalutamide was associated with a profound and more sustained PSA reduction compared to abiraterone, which translated into delayed disease progression. Both treatments were well tolerated, although a higher prevalence of adverse events was observed in the apalutamide cohort.

Low PSA levels during initial treatment have shown significant correlations with improved survival and clinical outcomes [11, 12, 20]. PSA50, PSA90, PSA ≤ 0.2 and PSA ≤ 0.02 rates were significantly higher in the apalutamide cohort at the first month follow-up compared to the abiraterone cohort. However, no statistically significant differences were observed in the other follow-ups regarding PSA50 and PSA90. Previous research had also observed no evidence of improved PSA90 rates at the 12-week follow-up in mHSPC patients between apalutamide, abiraterone and enzalutamide [14, 16], although Lowentritt et al. [15] did found a higher percentage of patients achieving PSA90 at 6- and 9-months follow-up in the apalutamide cohort. Moreover, apalutamide showed a higher proportion of patients achieving PSA levels ≤ 0.2 ng/ml and ≤ 0.02 than abiraterone at every time measured, also observed in previous observational studies where both apalutamide and enzalutamide showed similar results compared to abiraterone [14, 16], which was translated into a longer time remaining with ultralow PSA level. Although the abiraterone cohort had higher baseline PSA levels, similar results were observed in other studies with unbalanced PSA characteristics [14, 16] supporting the consistency of our findings. These results indicate that while both treatments are effective in reducing PSA levels, apalutamide achieves a deeper and more rapid PSA reduction, which is sustained for a longer duration compared to abiraterone, consistent with previous research.

In our study, the PSA nadir was significantly lower in the apalutamide cohort, with a median value of 0.02 [0.02–0.04] ng/mL, comparable to findings from both the TITAN trial population and other real-world studies [7, 14, 21]. Additionally, a shorter time to PSA nadir was observed in the apalutamide cohort. Wenzel et al. [13] observed that the proportion of mHSPC patients achieving a PSA nadir of ≤ 0.02 was significantly higher in the apalutamide cohort versus the abiraterone cohort. However, Lu Yutong et al. [14] patients did not find statistically significant differences in PSA nadir or time to nadir, although PSA values were lower on the apalutamide group, possibly due to the low number of patients included in this study.

Recent research indicates that achieving PSA levels below 0.02 ng/mL at any point during combination therapy confers substantial, statistically significant, and long-term benefits in OS and disease progression control [13, 22]. Furthermore, in a post-hoc analysis of the TITAN trial, PSA thresholds of ≤ 0.2 ng/mL and ≤ 0.02 ng/mL were associated with significantly longer rPFS, OS, time to PSA progression, and CRPC-PFS [12]. In our study, apalutamide showed a favorable PSA-PFS compared to abiraterone, demonstrating a significant advantage in delaying disease progression, consistent with other observational studies [13, 14]. Moreover, patients achieving PSA50, PSA90, PSA ≤ 0.2 and PSA ≤ 0.02 at any point during the study period as well as treatment with apalutamide appeared as protector factors in the univariate Cox regression analysis. However, only achieving ultralow PSA levels was associated with delayed progression in the multivariate analysis, having a 93% less risk of disease progression. Similarly, Wenzel et al. [13] observed that patients who achieved PSA nadir of ≤ 0.02 ng/mL with apalutamide had the best outcomes in terms of time to castration resistance and OS rates. Regarding OS, no statistically significant differences were observed between groups, as previously observed in other real-word studies [14, 16]. However, our OS results should be handled with care due to the low number of deaths and the influence of subsequent treatments, as can be seen in a higher percentage of abiraterone patients treated with docetaxel after progression, which could bias the results. Overall, these findings demonstrate a strong association between achieving ultralow PSA levels and delayed disease progression.

The safety profile of ARATs can also influence drug selection. A recent meta-analysis of randomised controlled trials assessing the adverse events associated with ARATs found a higher risk of high blood pressure and headache risk in patients treated with enzalutamide but no significant difference in adverse event rates between apalutamide and abiraterone [23]. However, in our study, a higher prevalence of adverse events was observed in the apalutamide cohort, with fatigue being the most common side effect, followed by skin rash. In a retrospective comparison of abiraterone, enzalutamide, and apalutamide, Suzuki et al. [16] reported a higher prevalence of grade 3 adverse events in the apalutamide cohort, with skin rash being the most frequent. Factors such as a broader and more heterogeneous population, more flexible monitoring and reporting standards, and variations in adherence could explain the differences observed between clinical trials and real-world data. Overall, both treatments were reasonably well-tolerated, with no adverse events leading to death and a low percentage of patients discontinuing treatment due to adverse events.

The findings of our study should be interpreted within the limitations of data and study design. Firstly, its single-centre and retrospective design restricts generalizability and may also introduce biases in data collection and reporting. Additionally, the retrospective nature limited our ability to prospectively power the study for several key endpoints (PSA kinetics, overall survival), potentially resulting in analyses underpowered to detect small but clinically meaningful effects. Secondly, considering the regression models for IPTW, it is important to highlight that unobserved baseline characteristics and confounders may remain unaccounted for, potentially leading to hidden imbalances in the index-treatment cohorts, even after applying IPTW. Moreover, baseline PSA was not fully balanced, which could result in an overestimation of the present findings. Thirdly, no data on dosage or dose reductions were available for inclusion in our analysis, which may have influenced the results. Despite these constraints, to our knowledge, this is the first study to provide an in-depth comparison of PSA dynamics between abiraterone and apalutamide treatments, addressing limitations in prior studies that either had very small patient cohorts, examined few variables, or did not adjust for baseline differences between groups, providing useful information for optimizing treatment strategies in mHSPC.

## Conclusion

Apalutamide showed a deeper and more sustained PSA reduction, which translated into delayed disease progression compared with abiraterone. Both treatments were well tolerated, although a higher prevalence of adverse events was observed in the apalutamide cohort. However, prospective randomised controlled studies with a larger population are needed to confirm the results of this observational study.

## Supplementary Information

Below is the link to the electronic supplementary material.

Supplementary file1 (DOCX 27 KB)
