# Supplementary material for: Real-world clinical outcomes of apalutamide versus abiraterone with androgen deprivation therapy for metastatic hormone-sensitive prostate cancer
Source: Int J Clin Pharm. 2025 May 6;47(6):1701–9. doi: 10.1007/s11096-025-01920-4 (PMC12630208; doi:10.1007/s11096-025-01920-4)
Supplement: Supplementary file 1 — Supplementary file1 (DOCX 27 KB) [file 11096_2025_1920_MOESM1_ESM.docx]

SUPPLEMENTARY FILES:


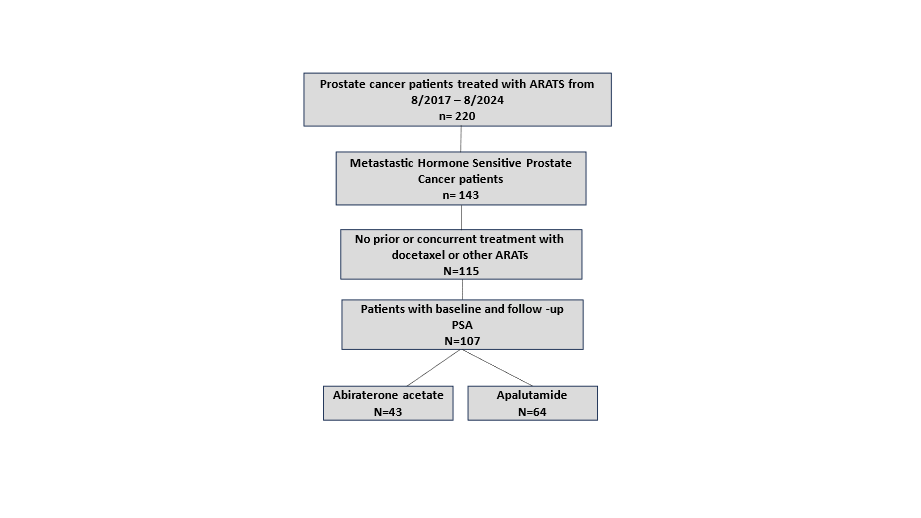


Supplementary Figure 1. : Patient flow diagram detailing the inclusion of study participants

| **Supplementary Table 1: Weighting Variables** |
| --- |
| Age |
| ECOG Peformance |
| Gleason Score |
| Prostatectomy |
| Concomitant radiotherapy |
| Metastasis Stage |
| Synchronous metastasis |
| Baseline PSA level |

| **Supplementary Table** **2. Univariate and multivariate COX regression analysis of selected variables for CRPC Progression-free Survival (CRPC-PFS)** | | | | |
| --- | --- | --- | --- | --- |
| **PSA-PFS** | **Univariate** | | **Multivariate** | |
|  | HR (95% CI) | p | HR (95% CI) | p |
| Metastasis stage, M1bc | 1.49 (0.9-2.6) | 0.410 | --- | --- |
| Gleason score, ≤7 | 0.34 (0.09-1.2) | 0.100 | --- | --- |
| Synchronous metastasis | 1.51 (0.63-3.7) | 0.350 | --- | --- |
| Baseline PSA level, ng/mL | 1.22 (0.9-1.6) | 0.066 | --- | --- |
| Radiotherapy | 1.32 (0.53-3) | 0.600 | --- | --- |
| PSA50 any time | 0.15 (0.04-0.5) | **0.002** | 0.94 (0.11-8.44) | 0.920 |
| PSA90 any time | 0.20 (0.05-0.67) | **0.009** | 0.41 (0.05-3.26) | 0.400 |
| PSA ≤0.2 ng/ml any time | 0.20 (0.08-0.48) | **<0.001** | 1.11 (0.31-4.01) | 0.900 |
| PSA ≤0.02 ng/ml any time | 0.07(0.02-0.23) | **<0.001** | 0.07 (0.02-0.28) | **<0.001** |
| Apalutamide | 0.36 (0.15-0.83) | **0.017** | 1.08 (0.30-3.09) | 0.913 |
| Abbreviations: CI: Confidence Interval; ECOG: Eastern Cooperative Oncology Group; HR: Hazard Ratio; PSA: Prostate-specific Antigen. | | | | |
|  | | | | |
